# Geospatial Analysis of Tsetse Infestation and Animal African Trypanosomiasis in Busia County, Kenya

**DOI:** 10.1155/japr/2532768

**Published:** 2026-07-18

**Authors:** S. S. Burudi, P. O. Mudavadi, J. K. Cheruiyot, E. O. Mungube

**Affiliations:** ^1^ Veterinary Science Research Centre, Trypanosomiasis Research Department, Kenya Agricultural and Livestock Research Organization (KALRO), Alupe, Busia, Kenya, kalro.org; ^2^ Department of Biological Sciences, Masinde Muliro University of Science and Technology, Kakamega, Kenya, mmust.ac.ke; ^3^ Livestock Health Research Division, KALRO Headquarters, Kenya Agricultural and Livestock Research Organization (KALRO), Nairobi, Kenya, kalro.org

**Keywords:** African animal trypanosomiasis, cattle, packed cell volume, prevalence

## Abstract

African trypanosomiasis (AT) is a vector‐borne disease caused by protozoan parasites of the genus *Trypanosoma*, primarily transmitted through the bite of infected tsetse flies in the genus *Glossina*. The disease is a major constraint to livestock production, causing reduced productivity, anemia, and mortality in affected animals. A cross‐sectional study was conducted from November to December 2024 to assess the spatial prevalence of AAT in cattle as well as tsetse fly abundance in Busia County, Kenya. A total of 762 cattle randomly selected from seven villages in Busia County were screened for AAT using the quantitative buffy coat technique. Packed cell volume (PCV) values were also determined. A tsetse survey was done by deploying 60 tsetse odor‐baited biconical traps in thickets and along streams in Funyula, Butula, and Teso North subcounties to estimate tsetse flies′ distribution and density. Overall reported prevalence of AAT was 6.82% (95% CI: 5.2–8.80). *Trypanosoma vivax* reported the highest prevalence amongst the reported species at 4.72%, which was significant (*p* = 0.006). Age, grazing system and breed significantly (*p* < 0.05) increased odds for trypanosome infection in cattle. Overall mean PCV for the study population was 24.82%. AAT‐positive cattle registered significantly lower PCV (22.7 ± 3.38%) compared with 26.94 ± 1.71.0% in the trypanosome‐negative cattle (*p* = 0.027). Two tsetse fly species were captured, with an apparent density ranging from 0.00 to 0.35 across the study sites. Species‐wise, tsetse catches were not statistically different in fly trap densities (FTD) between *Glossina pallidipes* (Gpd) and *Glossina fuscipes fuscipes* (*p* = 0.429), despite Gpd exhibiting a higher mean FTD (0.117, SD = 0.175). The study confirmed that AAT is still endemic in Busia County, with evidence that transmission is localized rather than widespread.

## 1. Introduction

Tsetse‐transmitted trypanosome infections of man and animals occur in 37 of the 54 countries in Africa, affecting an area of approximately 10 million square kilometers [1]. In humans, the disease is known as human African trypanosomiasis (HAT) or “sleeping sickness” (SS), whereas in domestic animals, the disease is known as Animal African Trypanosomiasis (AAT) or “nagana” [1]. Sleeping sickness occurs in two forms, gambiense HAT, which is responsible for the chronic form prevalent in West and Central Africa, and rhodesiense HAT (rHAT), which is the acute form prevalent in East and Southern Africa [2]. rHAT is zoonotic, and cattle serve as the sentinel domestic animal reservoirs [3]. An estimated population of 60 million people is at risk of SS in Africa, with an estimated 3 million people at moderate and high risk [3]. In Kenya alone, 7 million people are at risk of SS despite the elimination of the disease [4]. Sleeping sickness is a neglected disease with severe socioeconomic consequences in sub‐Saharan Africa and mainly affects poor people [5].

Nagana causes substantial livestock death and morbidity, thereby reducing the productivity of the sector in the region [1, 6]. Besides deaths of livestock, the disease causes direct losses by reducing meat and milk production, and indirect losses because of reduced fertility and draught capacity, and increasing the cost of livestock production. Estimates show that about 32% of the livestock population in sub‐Saharan Africa is found in tsetse‐fly‐infested areas [7], and it affects about 50 million heads of their cattle [8].

Furthermore, the disease has an extended impact on the crop, human settlement, and welfare, because 7 million square kilometers of sub‐Saharan Africa are rendered unsuitable for mixed crop‐livestock ecosystems [9]. AAT is estimated to cause annual losses of more than US$ 4.5 8 billion dollars through direct and indirect agricultural production costs [1]. It is not surprising that the 21 countries where trypanosomiasis is endemic are included in the world′s 25 poorest countries [10].

AAT is controlled mainly using chemotherapy although drug resistance has limited the effectiveness of this method. Trypanocidal drug resistance has been reported in at least 21 countries with the problem still expanding to other sub‐Saharan African countries [11–16]. Vector control is thus the only feasible option for controlling AAT and hence trypanocidal drug resistance [17]. The success of vector control in reducing trypanosomosis risk and resistance is documented in several reports[9, 18, 19].

The 1960s–1980s saw the introduction of odor‐baited traps and targets such as biconical and NGU traps, deployed in endemic areas like Siaya and Busia [20–22]. Several control programs, notably through the FITCA (1999–2004) and PATTEC (2005–2012) [23, 24], registered good successes in combating AT in western Kenya, notably in Busia, Siaya, and Bungoma. FITCA used a combination of tsetse fly traps, insecticide‐impregnated targets, insecticide‐treated cattle (ITC), and zero grazing systems and reduced tsetse populations by over 50% [25].

More recently, Kenya has adopted surveillance‐based approaches, including the development of a national tsetse atlas (2016–2019) by the Kenya Tsetse and Trypanosomiasis Eradication Council (KENTTEC) to guide interventions [26]. In 2020, the country implemented the Progressive Control Pathway (PCP), prioritizing endemic zones like western Kenya using geospatial data [26].

The country has also prioritized the eradication of HAT with a focus on the disease hotspots in Western Kenya. Consequently, KENTTEC has focused on suppressing the tsetse fly vectors to disrupt transmission of the disease to humans. As the control efforts are undertaken, active surveillance is done quarterly in areas covering Bungoma, Busia, and Siaya to monitor the effectiveness of the vector control efforts. During the surveillance visits, the focus is on the cattle as they are known sentinel reservoirs of *Trypanosoma brucei*, which causes HAT in humans. All positive cattle are treated to eliminate the parasite. Similarly, human cases that are positively diagnosed get treated. This study reports part of the findings of a surveillance conducted in Busia County. This study assessed the prevalence of AAT and the distribution of tsetse flies in selected areas of Busia County through a two‐phase survey combining entomological and parasitological assessments.

## 2. Materials and Methods

### 2.1. Study Area

The research was conducted in four subcounties of Busia County. The surveyed subcounties were Teso North, Teso South, Butula, and Funyula. Much of these areas are in the lowland wet ecosystems, which are suitable for the proliferation of *Glossina fuscipes fuscipes.* They also have thicket/bushes that are suitable for *Glossina pallidipes* [27]. Busia is a border county and has undergone substantial land use changes due to deforestation, urbanization, wetland reclamation, and livestock intensification, which could have potentially influenced vector ecology and AAT occurrence.

### 2.2. Selection of the Study Areas

**Figure 1:**
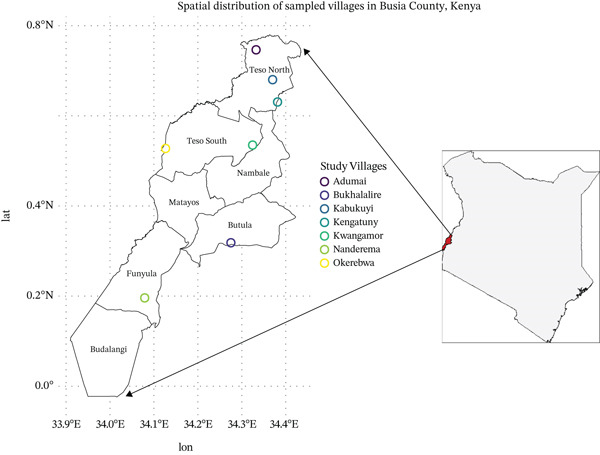
A map showing the distribution of villages where the AAT survey was conducted. Dotted circles represent the locations of the sampled villages, whereas labels indicate the corresponding subcounties in which the villages are located. The map illustrates the geographical coverage of the study area and the distribution of sampling sites across the county.

The study was done in seven villages in Busia County, which included Kabukuyi, Kengatuny, Nanderema, Kwangamor, Adumai, Bukhalalire, and Okerebwa (Figure 1). The study villages were selected purposively based on their known foci for HAT and have been historically mapped by PATTEC as such [28]. The villages are located along the porous Kenya–Uganda border presenting a high risk of tsetse fly invasion due to cross‐border livestock movement [29]. Biconical traps for tsetse flies were deployed in Teso North, Butula, and Funyula subcounties of Busia County.

### 2.3. Study Design

This was a cross‐sectional study conducted between November 1 and December 31, 2024, to estimate the prevalence of AAT and determine the density and distribution of tsetse flies in selected villages of Busia County, Western Kenya. The study was conducted during the short rain season to bring out seasonal influences on tsetse abundance in the region [30].

### 2.4. Sampling Design

A stratified random sampling approach was used to select bovines for AAT screening; each selected village was called a cluster, and a minimum of 69 animals were selected per village. The sample size calculation employed was *n* = (*Z* ^2^ *pq*)/*d* ^2^, a formula with the design effect formula of N = Nd for clusters, where *D* = 1 + (*m* − 1) ICC, and population characteristics between the previous studies and the current investigation [31–33], where

• *N*: population size,
• *Z*: *Z*‐score (critical value) at the desired confidence level,
• *p*: estimated sample proportion,
• *e*: margin of error,
• *Nd*: number of clusters selected,
• *D*: design effect; adjusts for clustering relative to simple random sampling,
• *ICC*: intracluster correlation coefficient; indicates similarity within clusters, and
• *M*: average number of individuals per cluster.

A total of 762 bovines were selected for screening of AAT across the study villages. A systematic sampling method was applied for trap deployment to assess tsetse fly density and distribution.

### 2.5. Sampling for Entomological Survey

An entomological survey was conducted along rivers and in bushes/thickets in three villages of Katotoi, Elukhari, and Nanderema in Busia County. Trap sites were selected using a stratified random sampling method to ensure representative coverage [34] and georeferenced with Garmin GPS units (±3‐m accuracy) [35]. Trapping was done along the riverine and open bushes. Protective wear was provided to the technical trapping team, with basic first aid kits carried along during the trapping exercise [36]. In total, 60 odor‐baited biconical traps were deployed across the selected villages. All the trapping sites were georeferenced by a Garmin GPS [33].

### 2.6. Trypanosome Prevalence

Each of the 762 selected cattle was bled and the blood examined for the presence of trypanosomes. Briefly, capillary blood was collected from every animal via ear pricks using venoject needles into heparinized capillary tubes [37]. The needle was used only once to avoid cross‐infection. Collected blood was carefully labelled before examination at the point of collection. The blood was evaluated for packed cell volume (PCV) using the Hawksley microhaematocrit reader method [38]. Microscopy was done using the buffy coat technique (BCT) for trypanosome detection [39, 40]. *Trypanosoma* spp. were morphologically identified based on their motility characteristics [41].

### 2.7. Tsetse Fly Distribution and Density

A total of 20 odor‐baited biconical traps were deployed in each selected village within historical SS foci [28, 42, 43]. After deploying the traps, they were retrieved after 48 h. Each trap had the number of flies counted before they were identified into their different species using morphological keys considering wing venation, antennal structure, body size, color, and geographical distribution [44]. This was used to estimate flies per trap per day and assess tsetse fly density based on studies indicating optimal capture rates without significant trap saturation [42]. Flies were then sorted based on feeding status [45].

### 2.8. Data Analysis

Data analysis involved both parasitological and entomological datasets. The prevalence of AAT was calculated as the proportion of infected cattle among the total examined, with 95% confidence intervals generated using OpenEpi. Differences in prevalence across villages were assessed using a multivariate analysis test, whereas Student′s *t*‐test compared mean PCV values between infected and noninfected cattle. One‐way ANOVA was used to assess variation in PCV across different *Trypanosoma* sp. Entomological data were analyzed by calculating the apparent density of tsetse flies as FTD, with the Kruskal–Wallis test used to compare fly densities between species. To explore the relationship between FTD and AAT prevalence in the county, Spearman′s rank correlation was computed. All analyses were performed using RStudio (Version 4.4.2), with statistical significance set at *p* < 0.05.

## 3. Results

### 3.1. Trypanosome Prevalence

**Figure 2:**
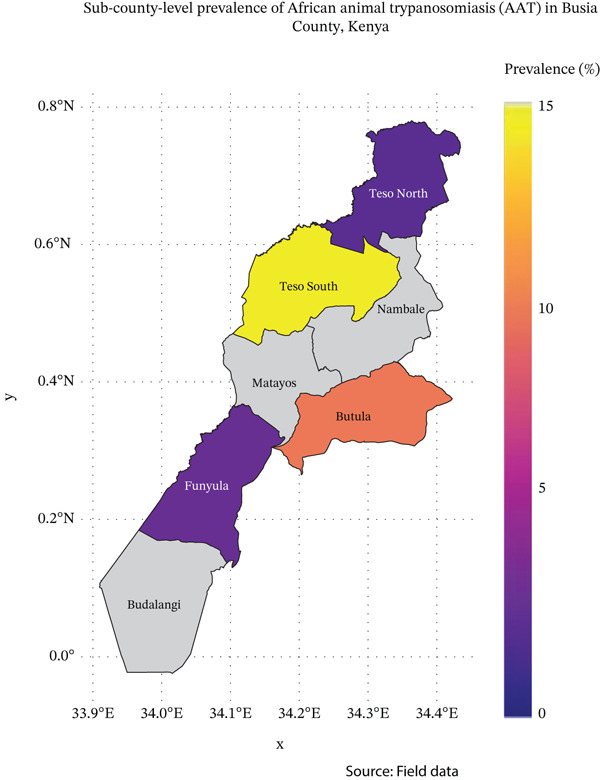
AAT spatial prevalence map from the cattle of Busia County (color intensity depicts prevalence of AAT in a given region).

The prevalence of trypanosomes varied across the seven villages ranging from 0 to 17.7% (95% CI: 11.1, 24.5) as shown in Table 1. Although there is no clear pattern, the prevalence depicted a southwest–northeast gradient as it increased from Nanderema in the south to Okerebwa and Kwangamor in the north (Figure 2). The overall prevalence of trypanosome infection in the sampled cattle (*n* = 762) was 6.82% (95% CI: 5.2–8.8). There was a statistically significant difference in trypanosome species prevalence (Kruskal–Wallis *χ* ^2^ = 10.11, df = 2, *p* = 0.006). Overall across the study villages, *Trypanosoma vivax* was the most prevalent with a general prevalence of 4.72%. A mean PCV of 25% was reported in cattle of Busia County.

**Table 1:** Trypanosome prevalence in cattle in seven villages of Busia County.

Village	Trypanosome‐positive cattle				Cattle examined	% Prevalence (95% CI)	% Mean PCV
	T. C.	T. V.	T. B.	Total			
Kabukuyi	0.92	2.75	0	4	109	3.7 (1.0, 9.1)	25.14
Kengatuny	1.45	2.9	0	3	69	4.4 (0.9, 12.2)	25.57
Nanderema	0.73	2.19	0	4	137	2.9 (0.8, 7.3)	28.70
Kwangamor	1.16	8.14	1.16	9	86	10.5 (4.9, 18.9)	24.14
Adumai	0	0	0	0	135	0	28.0
Bukhalire	3.3	6.59	0	9	91	9.9 (4.6, 8.0)	23.09
Okerebwa	5.93	11.11	0	23	135	17.0 (11.1, 24.5)	22.31
Total	1.97	4.72	0.13	52	762	**6.82 (5.2–8.8)**	25

Abbreviations: CI = confidence interval; PCV = packed cell volume; T. B. = *Trypanosoma brucei*; T. C. = *Trypanosoma congolense*; T. V. = *Trypanosoma vivax*.

### 3.2. Herd and Within‐Herd AAT Prevalence

AAT affects approximately one in five herds with an average proportion of infected animals within the affected herd moderately higher. The overall herd‐level prevalence was 19.0% (95% CI: 14.2–24.8), whereas the mean within‐herd prevalence among the 41 positive herds was 22.6% (95% CI: 17.6–28.5) (Table 2).

**Table 2:** Herd‐level and within‐herd prevalence of African trypanosomiasis by village in Busia County.

Village	No. of herds	No. of positive herds	Herd‐level prevalence (%) 95% CI	Total animals in positive herds	Total positive animals	Within‐herd prevalence (%) 95% CI
Kabukuyi	33	3	9.1(3.1–24.0)	13	4	30.8 (12.4–58.0)
Kengatuny	24	3	12.5(4.3–31.4)	12	3	25.0 (8.9–52.4)
Nanderema	33	4	12.1(4.8–27.3)	54	4	7.4 (2.9–17.2)
Kwangamor	24	4	16.7(6.7–35.0)	43	9	20.9 (11.4–35.2)
Adumai	29	0	0.0	0	0	—
Bukhalalire	38	10	26.3(14.8‐42.3)	30	9	30 (19.2–51.2)
Okerebwa	35	17	48.6(32.8–64.4)	78	23	29.5 (19.5–38.9)
Total	216	41	19.0 (14.2–24.8)	230	52	22.6 (17.6–28.5)

*Note:* Herd was described in this study as cattle presented by a single farmer, and within‐herd prevalence was calculated as the number of positive animals in a single herd as a percentage.

### 3.3. Effects of AAT on PCV

**Figure 3:**
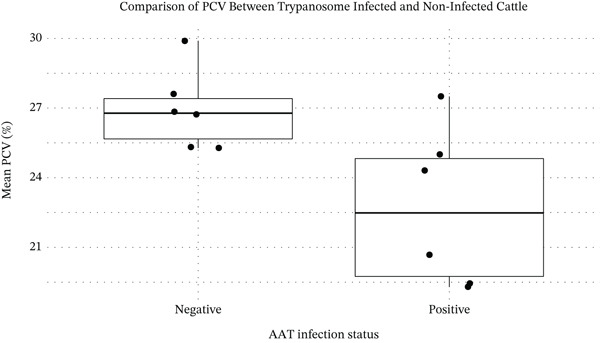
PCV values between AAT‐infected and noninfected cattle in Busia County. AAT‐positive cattle persistently demonstrated lower PCV values than noninfected cattle across the study sites in Busia County.

Mean PCV was significantly lower in trypanosome‐infected cattle (22.70 ± 3.38%) compared with noninfected cattle (26.94 ± 1.71%) Figure 3. An independent samples *t*‐test (Welch′s correction) demonstrated that this difference was statistically significant (*t* = 2.74, df = 7.40, *p* = 0.027). The mean reduction in PCV associated with infection was approximately 4.24 percentage points (95% CI: 0.62–7.86), indicating a substantial anemic effect of trypanosome infection. Cattle infected with *Trypanosoma congolense* exhibited the lowest PCV levels (20.5 ± 3.8); however, the difference was not statistically significant (*p* = 0.411) compared with cattle infected with *Trypanosoma vivax* and *Trypanosoma congolense* Table 3.

**Table 3:** Effect of different trypanosome species infection on PCV values of infected cattle.

ANOVA					
PCV					
	Sum of squares	df	Mean square	*F*	Sig.
Between groups	25.074	2	12.537	0.906	0.411
Within groups	678.002	49	13.837		
Total	703.077	51			

### 3.4. Risk Factors of AAT in Cattle in the Study Villages

Infection with trypanosomes was influenced by different risk factors. Age, grazing system, and breed significantly (*p* < 0.05) increased odds for trypanosome infection in cattle Table 4. Although female cattle had slightly higher odds of trypanosome infection than male ones, this was not significantly (*p* = 0.787) different.

**Table 4:** Risk factors of nagana in cattle of Busia County.

Risk factor	Variable	*n*		% Prevalence (95% CI)	Odds ratio	*p* value
Sex	Male	338	24	7.1 (4.78–10.39)	0.925	
	Female	424	28	6.6 (4.58–9.41)	1.081	0.787

Breed	Indigenous	687	45	6.6 (4.92–8.67)	1.0	
	Crossbreed	63	4	6.3 (2.05–15.67)	1.03	0.95
	Exotic	12	3	27.3 (9.2–57.1)	5.35	0.007

Grazing system	Free range	616	26	4.2 (2.87–6.14)	1.0	
	Private land	135	23	17.0 (11.57–24.33)	4.66	0.0001
	Zero grazing	11	3	27.3 (9.21–57.1)	8.47	0.023

Age group	0–24 months	171	7	4.1 (1.84–8.36)	0.041	0.03
	25–48 months	444	41	9.23 (6.86–12.31)	1.0	
	49–72 months	147	4	2.72 (0.83–7.02)	0.275	0.009

### 3.5. Tsetse Apparent Density and Distribution in Busia County

A mean tsetse FTD of 0.133 was reported across the study sites. Katotoi village in Teso North recorded the highest apparent density, FTD = 0.35 (7/8), whereas Nanderema had an apparent density of 0.05 (1/8), and no tsetse flies were reported in Elukhari Table 5. Two tsetse fly species were captured during the study*. Glossina pallidipes* with an FTD of 0.117 constituted 87.5% of total catches, higher than *Glossina fuscipes fuscipes* with an FTD of 0.017; this difference was not significant (Kruskal–Wallis: *χ* ^2^ = 1.90, df = 1, *p* = 0.168). Habitat‐based stratification indicated a higher population of tsetse flies in thicket habitats (FTD = 0.35) compared with the riverine environment (FTD = 0.025); this difference was statistically significant (Kruskal–Wallis: *χ* ^2^ = 5.40, df = 1, *p* = 0.020).

**Table 5:** Tsetse fly apparent density and distribution in Busia County, Kenya.

Area	No. of traps	Total flies captured	Fly species	Identification of fly sex and teneral and nonteneral statuses				Apparent fly density (FTD)
				NTM	NTF	TM	TF	
Katotoi	20	7	GFF	1	2	0	4	0.35
			GPD	0	0	0	0	0

Nanderema	20	1	GFF	0	0	0	1	0.05
			GPD	0	0	0	0	0

Elukhari	20	0	GFF	0	0	0	0	0
			GPD	0	0	0	0	0

Total	60	8		1	2	0	5	0.133

Abbreviations: FTD = fly per trap per day; GFF = *Glossina fuscipes fuscipes*; GPD = *Glossina pallidipes*; NMT = nonteneral male; NTF = nonteneral female; TF = teneral female; TM = teneral male.

### 3.6. Distribution and Abundance of Biting Flies

Biting flies were recorded opportunistically during the entomological survey across all study villages. *Stomoxys* spp. were the most captured (27 flies), and it was reported across the study sites. Other biting fly taxa, including members of the family Tabanidae and *Haematopota* spp., were present in lower numbers as summarized in Table 6.

**Table 6:** Biting flies abundance and distribution.

Subcounty	Village	Stomoxys (*n*)	Tabanidae (*n*)	Haematopota (*n*)	Total biting flies (*n*)
Funyula	Nanderema	9	1	1	11
Teso North	Katotoi	4	1	0	5
Butula	Elukhari	14	1	0	15
Total		27	3	1	31

## 4. Discussion

Findings from this study revealed that nagana is present in cattle in Busia County at a prevalence of 6.82%. This prevalence is higher than the reported national average of 1.3% [26] but is consistent with the prevalence (4%–9%) reported in studies conducted within ecologically similar settings located within the tsetse fly belt in Kenya [46–48]. The observed disparity between the current study and the national average may be attributed to differences in ecological conditions, vector abundance, livestock management systems, and study design used [26, 49]. A study in Lambwe Valley reported a prevalence of 3.3% using microscopy, which increased to 15.63% when more sensitive molecular diagnostics were applied [50]. Because the current study relied on microscopy, the prevalence of nagana in Busia County is likely underestimated and could be higher [51]. This high prevalence may be associated with persistent tsetse and mechanical vectors infestation, resistance to trypanocidal drugs, and frequent livestock movement across the county, with cross‐border movements being a possibility [14, 52, 53].

The study identified three *Trypanosoma* species infecting cattle in the region as *Trypanosoma vivax*, *Trypanosoma congolense*, and *Trypanosoma brucei*. All these species have previously been reported at both regional and national levels [26, 47]. *Trypanosoma vivax* is the most prevalent of the species, followed by *Trypanosoma congolense*, and *Trypanosoma brucei* occurring at the lowest frequency. This pattern has been consistently observed across the study sites and in other studies conducted within the western region and other parts of the country and neighboring countries [26, 46, 47, 50, 54, 55]. The predominance of *Trypanosoma vivax* is likely due to its ability to be transmitted not only by tsetse flies but also mechanically by biting flies such as *Stomoxys*, *Haematopota*, and *Tabanus* [56–58]. However, contrasting patterns have been reported in Lambwe Valley [59], southeastern Mali [14], and northwestern Ethiopia [60], where *Trypanosoma congolense* predominated. This may be explained by the consistent presence of biting flies in the study region of Busia County, unlike in Lambwe Valley, regions of Congo, and Ethiopia, where tsetse densities are consistently higher [27, 61]. Across these studies, *Trypanosoma brucei* consistently occurred at low prevalence, suggesting limited circulation within the cattle population and indicating a correspondingly low zoonotic risk for HAT in the region [2].

Anemia remains one of the most prominent clinical outcomes of trypanosome infections in cattle, typically reflected by a decrease in hematocrit [14, 62]. This study reported a lower PCV in infected animals compared with noninfected animals, confirming the expected association between trypanosomiasis and reduced red blood cell levels. Cattle infected with *Trypanosoma congolense* exhibited the lowest mean PCV compared with those infected with *Trypanosoma vivax* or *Trypanosoma brucei* [14, 62]. This trend suggests a potentially greater pathogenic effect of *Trypanosoma congolense*, which has also been observed in other endemic areas in Africa [59, 63]. The more pronounced anemia in *Trypanosoma congolense* infections is often linked to its higher parasitemia levels and longer persistence in host tissues [64, 65].

The study reported a low overall tsetse apparent density in Busia County, although variations were observed between villages and habitat types. The reported FTD of 0.133 is low compared with documented national FTD (3.5) [26] but similar to studies carried out in the Teso region of Busia County, which reported FTDs of between 0.08 and 1.5 [52]. This low FTD is because of sustained vector control efforts in the region involving tsetse fly trapping, deployment of insecticide‐impregnated targets, and cattle pour‐on techniques [66]. In addition, changing land use patterns through agricultural intensification and urbanization have interfered with tsetse breeding sites, thus reducing the tsetse fly population [67, 68]. Findings of the study revealed the presence of two tsetse fly species, namely, *Glossina pallidipes* and *Glossina fuscipes fuscipes*. These species have been reported before in the region [26, 52]. The study reports *Glossina pallidipes* dominant over *Glossina fuscipes fuscipes*; these findings are similar to the ones reported in the national atlas for tsetse in Kenya, where *Glossina pallidipes* constituted 87% of the total tsetse catches [26]. However, the study differs from the previous study in the same region by Adungo et al., who documented *Glossina fuscipes fuscipes* as the most dominant species [52]. This disparity can be explained by farmers′ persistent encroachment of water catchment areas for intensive and semi‐intensive farming, destroying the ideal breeding habitat for *Glossina fuscipes fuscipes* in the region [67–69].

The study identified breed, grazing system, and age as significant risk factors associated with trypanosome infection in cattle within Busia County, whereas sex was not significantly associated with infection. Similar observations have been reported in studies conducted in Tanzania [70], Ethiopia [71], and Lambwe Valley in Kenya [50, 59], where grazing and vector exposure patterns do not differ by sex. Study findings indicate that exotic and crossbreeds are more likely to be infected by AAT compared with the indigenous cattle. This study supports previous reports that local breeds possess greater tolerance or resilience to trypanosome infections under natural field conditions [50, 71, 72]. Exotic breeds are generally less adapted to endemic environments and may therefore be more susceptible to infection and disease establishment [26, 71].

The grazing system was also strongly associated with infection, with cattle maintained under private land and zero grazing systems showing higher infection levels compared with free‐range systems. The only limitation for these results was a small sample size of cattle under the zero grazing system presented for screening (11). These findings are similar to studies conducted in Ethiopia, where cattle under a tethering grazing system were more likely to be infected [72]. These findings differ from studies conducted in Lambwe Valley in Kenya, Tanzania, and Nigeria, which reported higher AAT prevalence in a communal grazing system, but the study sites were located near wildlife reservoirs [50, 73, 74]. These findings may be linked to localized vector concentration around restricted grazing and watering areas where animals repeatedly congregate, increasing the likelihood of contact with infected tsetse flies. In addition, agricultural land use practices and landscape fragmentation within Busia County may have created favorable microhabitats that sustain vector populations around livestock production units [27].

Age‐related variation in infection suggests that cattle within the productive age group are more exposed to infection than younger or older animals. Middle‐aged cattle are usually more active in grazing and herd movement, which may increase contact with tsetse‐infested environments. These results are similar to findings from studies conducted in Uganda [75], Ethiopia [71], and Lambwe Valley in Kenya [50]. In addition, tsetse flies have been reported to feed on adult cattle due to higher levels of kairomones (carbon dioxide, acetone, 1‐octen‐3‐ol, and phenols) released by cattle [26, 59, 76].

The study findings document a moderately high prevalence of AAT in cattle and a relatively low tsetse fly apparent density in Busia County. This finding contradicts historical studies in the region where AAT endemicity is heavily linked to a high tsetse fly population in the region [26]. Although this trend may initially seem counterintuitive, similar observations have been reported in other endemic regions like West Africa [77] and South Chad [78]. The declining apparent tsetse density, with the increasing AAT prevalence, can be attributed to the presence of secondary vectors, biting flies like Tabanus and Stomoxys, and potential transboundary animal movements [29, 57, 79]. Moreover, anthropogenic drivers such as deforestation, land conversion, and changing settlements in the region may have created localized hotspots for tsetse fly breeding [80].

## 5. Conclusion

AAT remains present in Busia County at a moderate level, with evidence that transmission is localized rather than widespread. The predominance of *Trypanosoma vivax* suggests that, in addition to tsetse flies, biting flies may be playing a key role in the mechanical transmission of nagana in the region. Infected cattle showed reduced PCV, confirming the negative effect of the disease on animal health and productivity under field conditions. The detection of *Glossina pallidipes* and *Glossina fuscipes fuscipes*, with higher densities in thicket habitats, indicates that environmental factors play an important role in maintaining vector populations. Overall, the findings show that AAT in the study area is sustained by a combination of ecological conditions, vector presence, and livestock management practices and continues to pose a constraint to livestock production.

To build on these findings, future studies should look more closely at the role of mechanical vectors in transmission. Incorporating molecular tools will also help improve the accuracy of diagnosing infections. Additionally, checking tsetse flies for trypanosome infections and examining their natural microbial communities could shed light on their ability to carry and spread the disease, helping refine control approaches.

## Funding

No funding was received for this manuscript.

## Conflicts of Interest

The authors declare no conflicts of interest.

## Data Availability

The data that support the findings of this study are available from the corresponding author upon reasonable request.
